# Individual animal variability in rumination, activity, and lying behavior during the periparturient period of dairy cattle

**DOI:** 10.3168/jdsc.2022-0300

**Published:** 2023-02-16

**Authors:** Ahmadreza Mirzaei, Victoria R. Merenda, Luiz F. Ferraretto, Randy D. Shaver, Francisco Peñagaricano, Ricardo C. Chebel

**Affiliations:** 1 Department of Large Animal Clinical Sciences, University of Florida, Gainesville 32608; 2 Department of Animal and Dairy Sciences, University of Wisconsin, Madison 53706; 3 Department of Animal Sciences, University of Florida, Gainesville 32608

## Abstract

The aim of the current study was to determine individual animal variability in rumination, activity, and lying behavior during the periparturient period within the context of dairy cattle nutrition, social, and physical environment. Holstein animals (nulliparous = 77, parous = 219) from one sand-bedded, freestall dairy in northwest Wisconsin were enrolled −17 d in milk (DIM, d 0 = calving), when they were fitted with an automated monitoring device (Hi-Tag, SCR Engineers Ltd.). At −11 DIM, animals were fitted with HOBO Pendant G Data Loggers. The HOBO Pendant G Data Loggers were fitted 6 d later because they were set up to collect data for 22 d (d −11 to 11), to avoid constant handling of the animals that could alter their behavior. Prepartum, nulliparous and parous animals were housed separately. Postpartum (1 to 17 ± 3 DIM), primiparous and multiparous cows were commingled. Samples of the total mixed ration were submitted for wet chemistry analysis and determination of physically effective NDF (peNDF). Temperature and humidity data were collected using RH Temp probes (HOBO Pro Series) installed in each of the pens, and the percentages of 30-min intervals within a day with temperature-humidity index ≥68 (PctTHI68) were calculated. Stocking density (cows per stall) during the pre- and postpartum periods were calculated daily. Prepartum data from nulliparous and parous animals were analyzed separately, and postpartum data from primiparous and multiparous animals were analyzed together. Prepartum, nulliparous and parous animals explained 83.9 and 64.5% of the variability in rumination, 70.7 and 60.9% of the variability in activity, and 38.1 and 63.6% of the variability in lying time, respectively. Postpartum, animal explained 49.7, 56.8, and 35.6% of the variability in rumination, activity, and lying time, respectively. Although stocking density, PctTHI68, peNDF, crude protein, and ether extract were associated with the variability in rumination, activity, and lying time, they explained ≤6.6% of the daily variability in these behaviors. We conclude that, within the conditions of the collaborating commercial herd, individual animal is the most important factor explaining daily variability in rumination, activity, and lying time.

## Highlights

• Automated behavior monitors are a practical tool for monitoring dairy cow behavior.
• Variability in peripartum behaviors is largely explained by the animal.
• Independent variables explained ≤6.6% of the variability in peripartum behaviors.
• To evaluate animal behavior, within-subject variation should be considered.
• Peripartum behavior assessment is difficult due to the range of individual variation.

## Graphical Abstract

**Figure:**
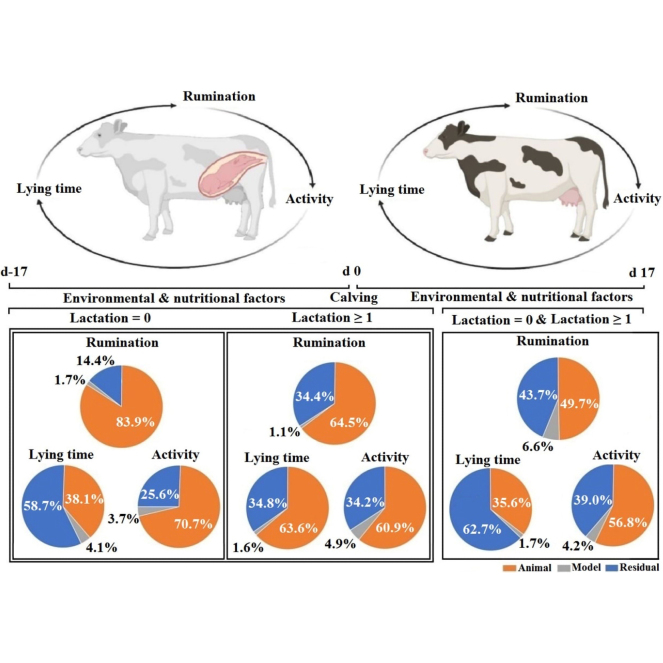
**Summary:** Peripartum behaviors (e.g., rumination, activity, lying time) of a large number of dairy cattle can now be monitored uninterruptedly. Such behaviors have been associated with peripartum health disorders and have been suggested to be indicators of poor management and inappropriate diets. We used automated devices to determine the individual animal variability in these behaviors before and after calving. Variability in rumination, activity, and lying behavior peripartum were largely explained by the animal (≥35.6%), whereas the contribution of environmental and nutritional variables to explain their daily variability was low (≤6.6%).

In recent years, automated monitors of behavior (e.g., rumination, activity, lying time) of dairy cattle have become more affordable, allowing for the constant monitoring of large populations of animals (Tucker et al., 2021). Some have hypothesized that changes in group averages of such behaviors may be indicative of failures in nutrition, environment, and management conditions. During the peripartum period, however, animals are generally housed in pens with constant (daily to weekly) regrouping. In addition to the effects of regrouping on behavior of prepartum (Schirmann et al., 2011; Lobeck-Luchterhand et al., 2014) and postpartum animals (von Keyserlingk et al., 2008), if individual animal largely explains the variability in such behaviors, the value of group averages to monitor nutrition, environment, and management conditions is questionable. Body size differences between breeds partly explains the variability in chewing and rumination behaviors (Aikman et al., 2008). Furthermore, the repeatability of rumination (Byskov et al., 2017), activity (Müller and Schrader, 2005), and lying time (Løvendahl and Munksgaard, 2016) within a lactation are moderate to high. This leads to the hypothesis that individual animal largely explains the daily variability in rumination, activity, and lying time.

Rumination, the time cows spend chewing their cud, serves the purposes of breaking down ingested feed to increase digestibility and generate bicarbonate. Physical properties of feeds affect passage rate, rumen function and digestibility. Physically effective NDF (**peNDF**) relates to the physical properties of fiber, which stimulates chewing and promotes the stratification of ruminal content into a floating mat over a pool of liquid and small particles (Mertens, 1997). Lying time is commonly associated with welfare because it is considered a highly valued resource (Munksgaard et al., 2005). Prepartum and postpartum cows spend 12 to 13 h/d lying down (Fregonesi et al., 2007; Lobeck-Luchterhand et al., 2015). Stocking density (Fregonesi et al., 2007; Krawczel et al., 2012; Lobeck-Luchterhand et al., 2015) and heat stress are negatively associated with lying time (Tresoldi et al., 2019). Thus, it is important to characterize individual animal variability in rumination, activity, and lying behavior in the context of nutrition and social and physical environments.

We hypothesized that the individual animal explains a large proportion of the daily variability of rumination, activity, and lying behavior during the periparturient period. As such, our objectives were to determine the individual animal variability in rumination, activity, and lying behavior in the context of nutrition and the social and physical environments.

Procedures were approved by the Institutional Animal Care and Use Committee, University of Minnesota. The current study was conducted in one dairy farm located in Rice Lake, Wisconsin, from August to December of 2013. The numbers of lactating and dry cows were 2,710 and 272, respectively, and 43% of adult cattle (lactation ≥1) were primiparous. The average rolling herd 305-d milk yield was 14,500 kg. Holstein animals (nulliparous = 77, parous = 219) were enrolled in the study −17 ± 3 DIM (d 0 = calving). Prepartum, nulliparous and parous animals were housed in separate pens (3 rows of stalls) in a naturally ventilated, sand-bedded freestall barn. Upon identification of signs of calving, animals were moved to a straw-bedded loose-housing pen. Postpartum (1 to 17 ± 3 DIM), primiparous and multiparous cows were commingled in one pen (3 rows of stalls) in a naturally ventilated, sand-bedded freestall barn. Stocking density (cows per stall) during the pre- and postpartum periods were calculated daily. Animals were enrolled in the study in weekly cohorts (n = 5 to 30).

Cows were fitted with automated monitoring devices (Hi-Tag, SCR Engineers Ltd.) from −17 ± 3 to 17 ± 3 DIM. Rumination and activity were recorded in minutes per 2-h interval, and rumination and activity data are presented as daily sum of all the intervals. Additionally, from −11 ± 3 to 11 ± 3 DIM cows were fitted with lying behavior loggers (HOBO Pendant G Data Logger, Onset Computer Corporation). Lying time was recorded every 1 min and was summarized to generate daily lying time.

During the study, TMR was offered once a day. Prepartum diets were formulated to meet or exceed the nutrient requirements of nonlactating Holstein cows weighing 610 (nulliparous) and 725 kg (parous), with conceptus gaining 0.6 kg/d and DM intake of 10 kg/d (NRC, 2001). Postpartum diets were formulated to meet or exceed nutrient requirements for lactating Holstein cows weighing 650 kg and producing 45 kg of 3.5% FCM (NRC, 2001). Diets were based on corn silage, alfalfa hay, soybean meal, steam-rolled corn, whole cottonseed, and a mineral and vitamin supplement mixture. Nutrient composition of diets is shown in Table 1.

**Table 1:** Composition of TMR offered to prepartum and postpartum cows

Variable	Prepartum				Postpartum	
	Nulliparous		Parous			
	Mean ± SD	Range	Mean ± SD	Range	Mean ± SD	Range
NE_L_, Mcal/kg	1.43 ± 0.03	1.39, 1.48	1.48 ± 0.02	1.39, 1.50	1.57 ± 0.04	1.45, 1.59
TDN, % of DM	63.0 ± 1.3	61.1, 64.6	64.7 ± 1.0	61.1, 65.9	68.8 ± 0.4	61.1, 69.8
NDF, % of DM	40.3 ± 0.9	39.3, 42.9	38.2 ± 1.8	35.2, 44.3	30.0 ± 0.4	29.3, 44.3
NFC, % of DM	30.9 ± 1.5	29.5, 34.1	35.6 ± 2.2	29.6, 38.7	41.0 ± 0.9	29.5, 41.9
CP, % of DM	16.6 ± 0.5	15.7, 17.9	16.0 ± 0.9	14.2, 18.0	18.5 ± 0.6	14.2, 19.8
Ether extract, % of DM	3.6 ± 0.4	3.1, 4.5	2.8 ± 0.14	2.5, 3.9	3.3 ± 0.3	2.7, 4.2
Ash, % of DM	9.5 ± 1.2	8.0, 11.1	8.1 ± 0.6	7.0, 11.0	8.3 ± 0.3	7.0, 11.0
Physically effective NDF, % of DM	30.5 ± 1.4	28.5, 33.3	27.5 ± 3.3	21.1, 37.3	15.9 ± 2.3	11.9, 37.2

As explained previously, nutritional, social, and environmental conditions affect rumination and lying behaviors. Thus, we collected information regarding diets, stocking density, and environment to control for them in the statistical analyses. Twice weekly, TMR from each of the pens were sampled (~1 kg) from 3 to 6 locations along the length of the feed bunk immediately after feed delivery. Samples collected from a pen within a week were homogenized manually and divided into 4 equal subsamples. Two subsamples allocated diagonally were remixed and sent to a commercial laboratory (Dairyland Laboratories Inc., Arcadia, WI) for analysis (AOAC International, 2012) of DM at 105°C (method 930.15), OM (method 942.05), CP (method 990.03), NDF (method 2002.04), ether extract (**EE**; method 2003.05). The NFC, TDN, and NE_L_ were calculated according to NRC (2001). The remaining 2 subsamples were analyzed separately for particle size distribution on an as-fed basis using the Penn State Particle Separator with 3 sieves (19, 8, and 1.18 mm; Heinrichs and Kononoff, 2002) and a bottom pan. Samples retained by each individual sieve were dried in a forced-air oven set at 60°C for 48 h and the proportion of particles retained on and above the 8-mm sieve calculated. The NDF content of the particles retained on and above the 8-mm sieve was determined as described previously. The peNDF was calculated as the proportion of TMR retained on and above the 8-mm sieve (DM basis) multiplied by the NDF content of that fraction. The average of the results from samples collected within week were used for each of the days within the corresponding week.

Temperature and humidity data were collected using RH Temp (HOBO Pro Series, Onset Computer Corporation) probes installed in each of the pens in which animals were housed. Temperature-humidity index was calculated for every 30-min interval within a 24-h period, and the percentage of 30-min intervals within a day with temperature-humidity index ≥68 (**PctTHI68**) was calculated for each pen.

All data were analyzed using SAS version 9.3 (SAS/STAT, SAS Institute Inc.). This was an observational study with animals enrolled in weekly cohorts of 5 to 30 animals. Continuous data were analyzed by ANOVA for repeated measurements using the MIXED procedure. Animal was included in the model as a random effect nested within pen, and day was the repeated measurement. Prepartum and postpartum data were analyzed separately. Calving day was excluded from the analyses. Prepartum data from nulliparous and parous animals were analyzed separately because they were housed in separate pens. Postpartum data from primiparous and multiparous cows were analyzed together because they were housed in the same pen. According to the Bayesian Akaike information criteria, autoregressive structure of covariance was chosen for all analyses. The associations of stocking density, PctTHI68, peNDF, CP, and EE and the outcomes of interest were tested using univariable models. Independent variables that, according to univariable analyses, had *P* < 0.20 were offered, as long as variance inflation factor was <2.5 according to a linear regression (REG procedure with collinearity and variance inflation factor functions). Ether extract presented collinearity with peNDF for postpartum rumination and activity, whereas CP and EE presented collinearity with peNDF for postpartum lying time. Nonfiber carbohydrate and NE_L_ were collinear with peNDF for all variables analyzed. Variables offered to the multivariable models were removed in a stepwise backward fashion so that only variables with *P* ≤ 0.10 were retained in the model. Statistical significance was defined as *P* ≤ 0.05, and tendencies were considered when 0.05 < *P* ≤ 0.10.

To calculate the percentage of variability explained by the individual animal and independent variables, 3 models were carried out: (1) no random effect and no independent variables (residual); (2) animal as a random effect nested within pen and no independent variables (animal); and (3) animal as a random effect nested within pen and independent variables (full model). The percentages of variability explained by the individual animal were calculated by dividing the intercept of the “animal model” by the residual of the “residual model.” The percentages of variability explained by the independent variables were calculated by dividing the subtraction of the intercept of the “full model” and “animal model” by the residual of the “residual model.”

During the prepartum period, the mean (±SD) stocking densities in the pens of nulliparous and parous animals were 86.2 ± 7.8 (range = 60.7, 102.7%) and 76.0 ± 10.2 (range = 50.0, 95.5%), respectively. The mean (±SD) PctTHI68 during the prepartum period were 28.5 ± 32.8% (range = 0.0, 100.0%) and 36.0 ± 35.8% (range = 0.0, 100.0%) in the pens with nulliparous and parous animals, respectively. In the postpartum period, the mean (±SD) stocking density was 72.8 ± 12.7 (range = 23.1, 100.9%), and the PctTHI68 was 30.1 ± 37.4% (range = 0.0, 100.0%).

Solutions for fixed effects retained in the multivariable models are presented in Table 2. For nulliparous animals, individual cow explained 83.9% of the variability in prepartum rumination, whereas the independent variables explained 1.7% of the variability in prepartum rumination. The association between PctTHI68 and rumination was quadratic because rumination time stayed relatively constant when PctTHI68 ranged from 0 to 40% when it started to increase. Physically effective NDF tended to be associated with rumination time such that the greatest rumination time was observed when peNDF was approximately 30%. For parous animals, individual cow explained 64.5% of the prepartum variability in rumination, whereas the independent variables explained 1.1% of variability in prepartum rumination. For every 1-unit increase in PctTHI68, we detected a 0.20-min/d decrease in rumination time. Furthermore, we detected a 1.3-min/d decrease in rumination time for every 1-unit increase in peNDF. The associations between CP and rumination time and EE and rumination time were quadratic. For both CP and EE, extreme values were associated with reduced rumination time.

**Table 2:** Solutions (estimates ± SE) for fixed effects retained in the final multivariable analyses

Variable	Prepartum						Postpartum		
	Nulliparous			Parous					
	Rumination	Activity	Lying time	Rumination	Activity	Lying time	Rumination	Activity	Lying time
Stocking^1^									
Linear	—	0.82 ± 0.23*	—	—	2.08 ± 1.12†	−1.17 ± 0.33*	0.49 ± 0.12*	—	5.15 ± 1.69*
Quadratic	—	—	—	—	−0.013 ± 0.007†	—	—	—	−0.035 ± 0.012*
PctTHI68^2^									
Linear	−0.30 ± 0.19	0.21 ± 0.07*	−1.10 ± 0.61†	−0.20 ± 0.05*	0.049 ± 0.090	0.57 ± 0.28*	−0.88 ± 0.12*	0.032 ± 0.117	−0.563 ± 0.122*
Quadratic	0.005 ± 0.002*	—	0.01 ± 0.01†	—	0.002 ± 0.001*	−0.008 ± 0.003*	0.007 ± 0.001*	0.005 ± 0.001*	—
peNDF^3^									
Linear	163.8 ± 89.1†	199.6 ± 58.9*	—	−1.27 ± 0.60*	—	−5.83 ± 1.54*	106.8 ± 11.0*	−20.9 ± 10.3*	82.0 ± 26.9*
Quadratic	−2.67 ± 1.44†	3.37 ± 0.95*	—	—	—	—	−3.74 ± 0.35*	0.87 ± 0.33*	−2.44 ± 0.81*
CP									
Linear	—	—	2,016.7 ± 650.7*	165.34 ± 83.63*	−5.20 ± 1.86*	—	−25.1 ± 4.7*	7.89 ± 4.39†	NT^4^
Quadratic	—	—	−60.0 ± 19.3*	−5.07 ± 2.59*	—	—	—	—	NT
Ether extract									
Linear	—	—	84.8 ± 28.2*	1,063.55 ± 401.30*	1,672.8 ± 282.6*	2,493.6 ± 995.9*	NT	NT	NT
Quadratic	—	—	—	−193.05 ± 71.71*	−284.5 ± 50.1*	−442.6 ± 171.7*	NT	NT	NT

1 Stocking density according to number of stalls.

2 Percentage of 30-min intervals within a day with temperature-humidity index ≥68.

3 Physically effective NDF.

4 NT = not tested because variance inflation factor ≥2.5.

† 0.05 < *P* ≤ 0.10.

* *P* ≤ 0.05.

For nulliparous animals, individual cow explained 70.7% of the variability in prepartum activity, whereas the independent variables explained 3.7% of the daily variability in prepartum activity. We detected that for every 1-unit increase in stocking density there was a 0.82-arbitrary unit (**AU**) increase in activity, whereas for every 1-unit increase in the PctTHI68 we detected a 0.21-AU increase in activity. We found a quadratic association between peNDF and activity such that activity remained relatively constant between 28.5 and 30.0% and increased thereafter. For parous animals, individual cow and the independent variables explained 60.9 and 4.9% of variability in prepartum activity, respectively. We observed a tendency for a quadratic association between stocking density and activity such that activity increased up to approximately 80%, when a plateau was reached. Similarly, PctTHI68 was quadratically associated with activity. Activity remained relatively constant when PctTHI68 increased between 0 and 40%, whereas it increased sharply when PctTHI68 was ≥45%. For every 1-unit increase in CP, we detected a 5-AU decrease in daily activity. Ether extract was quadratically associated with activity such that activity was greatest when EE was approximately 2.8%.

For nulliparous animals, individual cow explained 38.1% of the variability in prepartum lying time, whereas the independent variables explained 4.1% of the variability in prepartum lying time. We detected a tendency for a quadratic association between PctTHI68 and lying time such that lying time was lowest when PctTHI68 was approximately 50%. Similarly, CP was quadratically associated with lying time. Lying time increased as CP increased from 15.5 to 16.5%, when it reached a plateau. For every 1-unit increase in EE, we detected an 85-min/d increase in lying time. For parous animals, individual cow explained 63.6% and the independent variables explained 1.6% of the variability in prepartum lying time. Lying time decreased by 1.2 min/d for every 1-unit increase in stocking density. A quadratic association was detected between PctTHI68 and lying time. Lying time increased when PctTHI68 increased from 0 to 60%, when it reached a plateau. For every 1-unit increase in peNDF, we detected a 5.8-min/d decrease in lying time. The association between EE and lying time was quadratic, as the lowest lying time was associated with extremes of EE.

Animal and the independent variables explained 49.7 and 6.6% of the daily variability in postpartum rumination time, respectively. A 1-unit increase in stocking density was associated with a 0.5-min/d increase in rumination time. We detected a quadratic association between PctTHI68 and rumination time, such that rumination time decreased when PctTHI68 increased from 0 to 70%, when it reached a plateau. The association between peNDF and rumination time was quadratic, because rumination time was greatest when peNDF was approximately 15%. Rumination time decreased by 25 min/d for every 1-unit increase in CP. Animal and the independent variables explained 56.8 and 4.2%, respectively, of the daily variability in activity postpartum. The association between PctTHI68 and activity was quadratic. Similarly, peNDF was quadratically associated with activity such that it remained relatively constant from 12 to 15% and increased steeply thereafter. A 1-unit increase in CP tended to be associated with a 7.9-AU increase in daily activity. Postpartum, animal explained 35.6% of the daily variability in lying time, whereas the independent variables explained 1.7%. We found a quadratic association between stocking density and lying time, because lying time was greatest when stocking density was approximately 70%. Furthermore, lying time decreased by 0.6 min/d for every 1-unit increase in PctTHI68. The association between peNDF and lying time was quadratic such that extremes of peNDF were associated with the lowest lying time.

In the current study, we attempted to identify, within one dairy herd, the individual animal variability in rumination, activity, and lying time peripartum. As such, we are unable to establish cause and effect, and the external validity of our results is limited to conditions similar to those of the collaborating dairy. Nonetheless, the present study offers important insights regarding individual animal variability in these behaviors in a commercial dairy herd, in the context of their nutritional, social, and physical environments. We demonstrated that the animal explained 35.6 to 83.9% of the daily variability in rumination, activity, and lying time peripartum. Some have suggested that changes in group averages of such behaviors may be indicative of nutrition, environment, and management deficiencies. Our results suggest that evaluation of the variability of such behaviors in dynamic groups, as during the close-up period (last 21 d of gestation) and immediately postpartum (up to 21 d postpartum), is difficult because of the high degree of individual variation.

Others have demonstrated that behaviors such as rumination, eating time, activity, and lying time are heritable and repeatable. Byskov et al. (2017) demonstrated that repeatability of rumination time ranges from 0.83 to 0.94 from early to late lactation, whereas heritability ranges from 0.14 to 0.44. Müller and Schrader (2005) demonstrated that, within lactation, the repeatability of periods of high and low activity was between 0.40 and 0.62, corroborating our findings that activity is largely explained by the animal. Løvendahl and Munksgaard (2016) demonstrated that, within a lactation, repeatability of eating time ranged from 0.44 to 0.58, whereas repeatability of lying time ranged from 0.25 to 0.44. When comparing these behaviors during early and late lactation, the authors reported a high correlation, ≥0.76 (Løvendahl and Munksgaard, 2016). Together, these data reinforce the importance of using methods that account for within-subject variation when examining these behaviors.

Although not the main objective of the current study, we evaluated a few nutrition and environment variables associated with behavior. In the conditions of the current study, independent variables explained little of the variability in rumination, activity, and lying time during the periparturient period. It is worth noting that the ranges of CP, EE, and peNDF were narrow, limiting our ability to more clearly evaluate their contribution to daily variability in the behaviors evaluated. The association between peNDF and chewing activity is linear (Yang and Beauchemin, 2006). Although chewing time increased from 701.8 to 783.3 min/d when peNDF increased from 8.9 to 11.5% of DM (Beauchemin and Yang, 2005), when peNDF increases beyond 15% a linear decrease in DMI may be observed (Zebeli et al., 2010). The association between PctTHI68 and rumination was expected. A negative correlation between THI and rumination time has been reported (Soriani et al., 2013; Moretti et al., 2017); however, smaller animals are believed to be more heat tolerant because of their lower metabolic heat generation and higher ratio of surface area relative to internal body mass (West, 2003). This could explain the difference in the association of PctTHI68 and rumination time between nulliparous and parous animals. Stocking density was positively associated with activity of nulliparous and parous animals and negatively associated with lying time of parous animals. Housing prepartum cows at 80% stocking density resulted in fewer feed bunk displacements compared with 100% stocking density (Lobeck-Luchterhand et al., 2015), leading to the hypothesis that high stocking density results in greater competition for resources and consequently greater activity. In experiments with postpartum cows, increases in stocking density from 100% to ≥131% resulted in reduced percentages of cows lying (Hill et al., 2009) and decreased lying time (Fregonesi et al., 2007; Hill et al., 2009; Krawczel et al., 2012), which corroborates our findings.
